# Challenges of AI driven diagnosis of chest X-rays transmitted through smart phones: a case study in COVID-19

**DOI:** 10.1038/s41598-023-44653-y

**Published:** 2023-10-23

**Authors:** Mariamma Antony, Siva Teja Kakileti, Rachit Shah, Sabyasachi Sahoo, Chiranjib Bhattacharyya, Geetha Manjunath

**Affiliations:** 1 Indian Institute of Science, Bangalore, India; 2 Niramai Health Analytix, Bangalore, India

**Keywords:** Machine learning, Radiography

## Abstract

Healthcare delivery during the initial days of outbreak of COVID-19 pandemic was badly impacted due to large number of severely infected patients posing an unprecedented global challenge. Although the importance of Chest X-rays (CXRs) in meeting this challenge has now been widely recognized, speedy diagnosis of CXRs remains an outstanding challenge because of fewer Radiologists. The exponential increase in Smart Phone ownership globally, including LMICs, provides an opportunity for exploring AI-driven diagnostic tools when provided with large volumes of CXRs transmitted through Smart Phones. However, the challenges associated with such systems have not been studied to the best of our knowledge. In this paper, we show that the predictions of AI-driven models on CXR images transmitted through Smart Phones via applications, such as WhatsApp, suffer both in terms of Predictability and Explainability, two key aspects of any automated Medical Diagnosis system. We find that several existing Deep learning based models exhibit *prediction instability*–disagreement between the prediction outcome of the original image and the transmitted image. Concomitantly we find that the explainability of the models deteriorate substantially, prediction on the transmitted CXR is often driven by features present outside the lung region, clearly a manifestation of Spurious Correlations. Our study reveals that there is significant compression of high-resolution CXR images, sometimes as high as 95%, and this could be the reason behind these two problems. Apart from demonstrating these problems, our main contribution is to show that Multi-Task learning (MTL) can serve as an effective bulwark against the aforementioned problems. We show that MTL models exhibit substantially more robustness, 40% over existing baselines. Explainability of such models, when measured by a saliency score dependent on out-of-lung features, also show a 35% improvement. The study is conducted on **WaCXR** dataset, a curated dataset of 6562 image pairs corresponding to original uncompressed and WhatsApp compressed CXR images. Keeping in mind that there are no previous datasets to study such problems, we open-source this data along with all implementations.

## Introduction

The global outbreak of the COVID-19 pandemic had a severe impact on healthcare systems worldwide. During this time, there was a consensus among experts that Chest X-Rays (CXR) should be prioritized as a first-line triage tool for COVID-19 detection^1^. However, relying on CXR imaging for triaging placed a significant burden on the limited number of radiology experts in each hospital, especially in Low-to-Middle-Income countries (LMICs) where the ratio of radiologists to radiographers is significantly imbalanced. For example, in India, there is only one radiologist for every 100,000 people^2^. Compounding the issue is the fact that most radiologists are located in urban areas, which are often far from the majority of the X-ray imaging facilities located in rural areas.

One potential solution to this challenge is the development of a centralized AI-based system for COVID-19 detection that can assist radiologists in the interpretation of CXR images. However, a major hurdle in creating such an AI system is ensuring its technical simplicity, allowing doctors with minimal technical expertise to access and utilize it without additional support. This is crucial to minimize deployment costs, particularly in rural hospitals and clinics that may lack dedicated IT departments. As a result, the use of smartphone-based applications (henceforth referred to as Apps) is gaining prominence.

In recent years, smartphone penetration has significantly increased, especially in LMICs, with over 90% ownership and around 50% mobile internet connectivity^3^. This presents new opportunities for the quick and easy sharing of radiographic images to aid clinical decision-making^4^. Studies have shown that smartphone-captured radiographic images, communicated through messaging applications like WhatsApp, demonstrate similar diagnostic accuracy compared to viewing images on full-featured PACS systems^4^. In these approaches, a CXR image captured via smartphone or digital camera is transmitted to radiologists for remote review and diagnosis. However, manual processing of CXR images becomes impractical during a pandemic due to the large volume of data involved.

The combination of smartphones and AI apps for COVID-19 detection can enhance accessibility, particularly in rural areas. Such systems support patient triaging in resource-constrained settings and high-prevalence areas and alleviate the pressure on hospitals and healthcare workers. Throughout the pandemic, several such systems, known as AI-aided Diagnosis of X-ray images through messaging Applications (**AIDXA**), have been launched globally. Prominent smartphone applications, such as, XraySetu^5^, CAD4COVID^6^, LUNIT INSIGHT CXR^7^, and qXR^8^, address the challenges associated with low bandwidth in rural areas and facilitate ease of training. Some of these applications have utilized messaging interfaces like WhatsApp and Telegram for communication. For instance, Xraysetu was launched in India during the COVID-19 pandemic to provide AI interpretation over WhatsApp^9^. Leveraging common messaging platforms like WhatsApp eliminates the need for additional training as it is a widely used communication medium. However, it is important to note that these messaging platforms often compress images to overcome bandwidth limitations.

**Figure 1:**
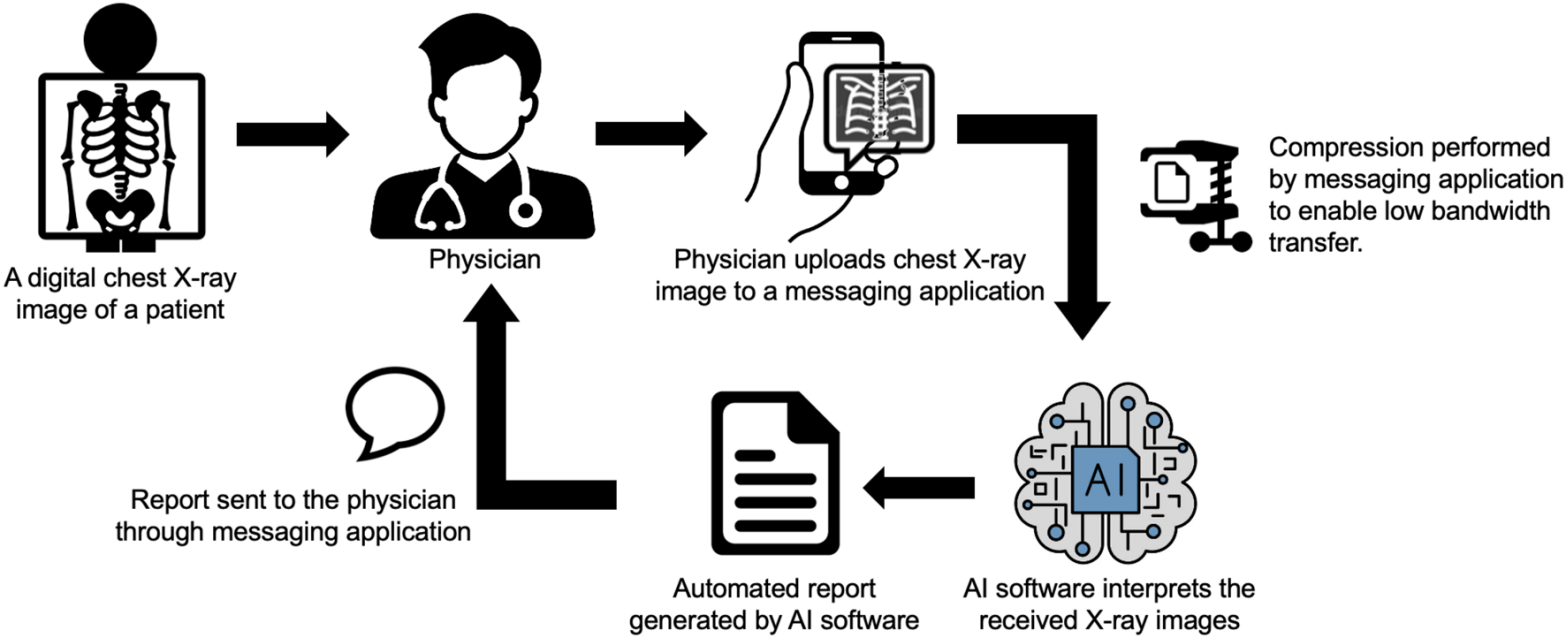
Schematic drawing of an AI aided Diagnosis of Xray images through messaging Applications (**AIDXA**) system. In this case study, we specifically study the challenges faced with the AI software in analyzing the X-ray images uploaded to a messaging application and propose modifications to the AI software to generate a reliable dignosis.

In summary, **AIDXA** systems are highly promising approaches for addressing the critical shortage of expert human radiologists. These approaches utilize WhatsApp or other similar apps as channels for transmitting CXR images. The communication process involves two steps: (a) a doctor shares the CXR images with the **AIDXA** system. (b) the system provides an automated report indicating the diagnosis through the chosen app. Figure 1 depicts a schematic diagram of a generic **AIDXA** system for AI aided Diagnosis of X-ray images through Apps.

### A case study

The pandemic unleashed by COVID-19 placed a severe burden on healthcare systems worldwide. During the pandemic several AI-based Chest X-Ray (CXR) imaging products were launched, driven by the exponential increase in smartphone ownership globally, especially Low and Middle-Income Countries (LMICs). Such AI-based products integrated with low-bandwidth messaging applications like WhatsApp facilitate the rapid triaging of infected individuals. To investigate the technical challenges associated with developing an AI-based Diagnosis of X-Ray Images through messaging Applications (**AIDXA**), a case study was conducted. The study utilized an existing open-source dataset of CXR images from COVID-19 patients to create a new paired dataset. This paired dataset consist of the original CXR images and the corresponding WhatsApp-transmitted images. Several state-of-the-art Deep Learning Networks for diagnosing COVID-19 images were employed to compare the diagnosis on the original image with that on the paired image. This case study identified two primary challenges that arise when deploying **AIDXA**: *prediction instability* and *out-of-lung saliency*. These challenges are briefly described below.

### Prediction instability (**PIP**)

**PIP** refers to a situation where a prediction model exhibits instability when diagnosing a CXR image, resulting in disagreement between the model’s predictions on the original image and the image transmitted through WhatsApp. Experiments conducted on the COVID-Net dataset, which consists of open-source COVID-19 CXR images, using various state-of-the-art models (ResNet^10^, ResNeXt^11^, XceptionNet^12^, VGG-19^13^, COVID-Net^14^) revealed a significant portion of examples affected by **PIP**. This undesirable effect can have critical implications in medical applications like the one being considered. While some recent studies^15,16^ have addressed the problem of model robustness and generalization through pixel perturbation in image classification tasks, the issue of **PIP** resulting from distortions in **AIDXA** systems has not been thoroughly explored in the literature.

### Out of lung saliency (OLS)

**Figure 2:**
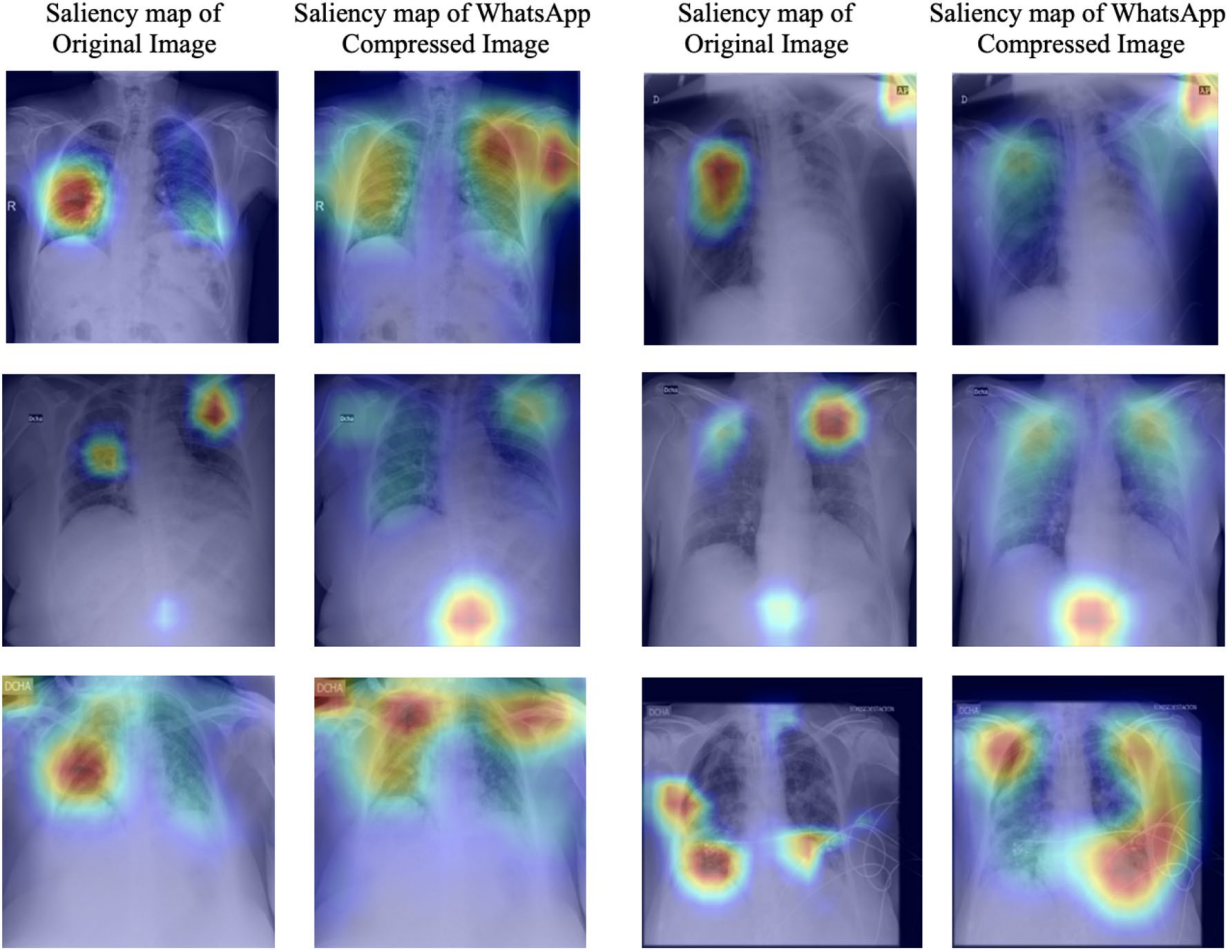
Exemplified COVID-19 localization generated with ResNet and ResNext architectures on (**a**) original images and (**b**) their corresponding WhatsApp compressed images from **WaCXR** dataset. Though the images look visually similar after compression, the predictions and localizations generated by deep learning architectures can significantly differ.

Existing literature^17^ suggests that the high predictive accuracies obtained by Deep learning models can often be attributed to learning unintentional *shortcut* strategies or spurious features. In medical diagnosis, it is crucial to provide explainable predictions. Saliency maps, which identify localized regions in an image that significantly contribute to the prediction, are commonly used to obtain explanations. Among other things, saliency maps help identify major confounding factors between spurious features^18^ and pathology. Figure 2 displays several instances of saliency maps generated by state-of-the-art models on original and corresponding WhatsApp-compressed COVID-19 CXR image pairs. It is evident that in all of these images, saliency maps highlight regions outside the lungs as significant contributors to the prediction. This effect is even more pronouced in WhatsApp-compressed images. In the context of CXR-based COVID-19 detection, *out-of-lung saliency* (**OLS**) can be considered a manifestation of spurious features. The introduction of WhatsApp exacerbates the impact of **OLS** as illustrated in Fig. 2.

### Contributions

The case study aimed to understand the technical challenges associated with **AIDXA** systems discovers that state-of-the-art Deep Networks integrated with low-bandwidth messaging applications suffer from several problems not reported in the literature. Furthermore, the case study demonstrates that Multi-Task Learning can effectively address the aforementioned problems. A summary of the key contributions are as follows.

- The paper demonstrates that existing state-of-the-art Deep Networks are susceptible to the problems of **PIP** and **OLS** when integrated with low-bandwidth messaging applications like WhatsApp. **PIP** and **OLS** can be viewed as instances of spurious correlations in Deep Networks for diagnosing CXR images. While the notion of spurious correlations exist in the literature, this paper uncovers the two instances of spurious correlations: **PIP** and **OLS**. A quantitaive characterization of both these problems are provided. Although several works in the literature point out the occurrence of spurious correlations in CXR images, this is the first work that defines metrics to quantify instances of spurious correlations to the best of our knowledge. While these challenges can be mitigated by training neural networks on WhatsApp-compressed CXR datasets, this approach may introduce bias and may not generalize well to other **AIDXA** systems. Therefore, the reliable diagnosis without the need for specific **AIDXA** datasets remains an open problem.
- The paper demonstrates that Multi-Task learning (MTL) framework can mitigate the effect of **PIP** and **OLS** . The paper proposes a *Multi-Task learning (MTL)* framework for COVID-19 detection called **COVIDMT**, trained on high resolution CXR images from patients with various lung abnormalities to classify COVID-19. The quantitative metrics developed in the paper demonstrate that applying the MTL framework to a benchmark dataset reduces the impact of **PIP** and **OLS** by an average of 40% and 35%, respectively, compared to base models such as ResNeXt^10^ and ResNet^11^. Although MTL model have been used in the literature for COVID-19 diagnosis, this is the first attempt to demonstrate that model trained using multiple datasets simultaneously attains a natural form of generalization to mitigate the effect of **PIP** and **OLS**.
- To facilitate the benchmarking of Deep Learning Models on CXRs transmitted through **AIDXA** systems, the paper open-sources the **WaCXR** dataset. This dataset comprises 6562 pairs of high-resolution and low-resolution CXR’s, making it the largest medical dataset generated using WhatsApp to the best of our knowledge.

The paper is structured as follows: First, there is the related works section which provides an overview of Deep Learning Networks for COVID-19 diagnosis and multi-task learning. The next section, titled Materials and Methods, describes the datasets and discusses the problems of **PIP** and **OLS** highlighted in this case study. This section also defines metrics to quantify the **PIP** and **OLS** of state-of-the-art models. The subsequent section describes the multi-task learning approach employed in this case study, named **COVIDMT**, which serves as an effective bulwark against **PIP** and **OLS**. The experiments section discuss the performance of **COVIDMT** and compares the robustness of **COVIDMT** with state-of-the-art models. Finally, there is the Discussion and Conclusion section.

## Related work

### Deep learning based COVID-19 detection

Several studies have proposed deep learning frameworks for the automatic detection of COVID-19 using CXR images. Bressem et.al^19^ compares the performance of several pre-trained models (ResNet^10^, DenseNet^20^, VGG^13^, and Inception v4^21^) for training on the classification of COVID-19 and normal CXR images. Narin et al.^22^ proposed an automatic detection method using five pre-trained convolutional neural network-based models, with the ResNet50 model demonstrating the highest performance. Similar works^23–25^ employ transfer learning on existing models such as ResNet^10^, VGG^13^, DenseNet^20^, and MobileNet^26^. COVIDNet^14^ is a Deep Learning Network designed from scratch for predicting COVID-19 using CXR images. Afshar et al.^27^ proposed COVID-CAPS, an alternative modeling framework based on Capsule Networks, capable of handling small datasets and achieving high accuracy and specificity. Zhang et al.^28^ propose B-DDLN, which consists of a feature extractor made of convolution blocks followed by a bagging classifier made of *N* dynamic learning networks. Stubblefield et al.^29^ investigate the utility of Deep Neural Networks as feature extractors for classical networks such as XGBoost to be applied to smaller datasets. Bayram and Eleyan^30^ proposed a cost-effective method using a multi-stream convolutional neural network model that extracts and concatenates features from multiple image types. De Falco et al.^31^ proposed a new general-purpose method that relies on interpretability ideas, using a filtering scheme and an evolutionary algorithm to automatically extract explicit knowledge in the form of IF-THEN rules.

### Challenges with deep learning based COVID-19 detection

Most of the proposed approaches either do not analyze the explainability or examine explainability by providing saliency maps of a few CXR images. According to Chowdhury et al.^32^, one challenge in using Deep Learning algorithms to detect COVID-19 from CXRs is distinguishing it from other viral pneumonia. This is due to the similarity and overlap of their images with other infectious and inflammatory lung diseases. Hall et al.^33^ faced challenges in their study, including a lack of information about the disease stage in COVID-19 cases and a small data set size. Maghdid et al.^34^ describe the lack of a publicly available dataset of CXR and CT images as a challenge. Zheng et al.^35^ addressed the need for annotated data by developing a weakly-supervised Deep Learning-based software system using 3D CT volumes. This system can accurately predict the COVID-19 infectious probability in chest CT volumes without requiring lesion annotations for training.

Sadre et al.^36^ analyses the accuracy and sensitivity of Deep Learning Models (CovidNet-CXR3-A^14^, CovidNet-CXR4-A^14^, VGG-11^13^, ResNet^10^) that are trained on CXR dataset by including and excluding lung region separately. Sadre et al.^36^ observe that the model trained by excluding the lung region of CXR image gives a similarly high accuracy when compared with a model trained by using only the lung region of a CXR image. DeGrave et al.^18^ show that many state-of-the-art Deep Learning Models learn spurious correlations between presence/absence of COVID-19 and radiographic features. Daniel et al.^37^ also points out the issue of “shortcut learning” in most of the Deep Learning Models and highlights that such models fail to learn the true underlying pathology reflecting the presence of COVID-19; instead leverage biases in the dataset, that are not directly related to pathologies associated with images. These works highlight the need to verify that AI systems rely on desired signals for the prediction of COVID-19.

### Model explainability for COVID-19 detection

Singh et al.^38^ proposed a novel Deep Learning-based solution using CXRs for rapid triaging of COVID-19 patients, incorporating explainability through the use of Grad-CAM visualization to establish trust in the medical AI system. Chetoui et al.^39^ developed a Deep Learning algorithm to detect COVID-19, pneumonia and normal cases on CXR images, using an explainability algorithm to visually show the location of the lung-infected areas detected by the model. Wang et al.^14^ introduced COVID-Net, a deep convolutional neural network design for detecting COVID-19 cases from CXR images, and investigated its predictions using an explainability method to gain deeper insights into critical factors associated with COVID-19 cases and to audit the model in a responsible and transparent manner. Gidde et al.^40^ developed CovBaseAI, an explainable tool using an ensemble of three Deep Learning Models and an expert decision system (EDS) for COVID-Pneumonia diagnosis, trained entirely on pre-COVID-19 datasets, providing new insights on the usage of EDS with Deep Learning methods for confidently predicting COVID-Pneumonia. Barbano et al.^41^ proposed a two-step diagnostic approach for Covid-19 detection from CXR images, designed to mimic the diagnosis process of human radiologists, providing structural explainability to build trust between physicians and AI models.

### Spurious correlation of deep learning algorithms based COVID-19 detection on CXRs

Wynants et al.^42^ conducted a systematic review and critical appraisal of prediction models for COVID-19 prognosis and risk detection, finding that most published models were poorly reported and at high risk of bias. Ghoshal and Tucker^43^ showed that the uncertainty in prediction is highly correlated with the accuracy of prediction and proposed Bayesian Convolutional Neural Networks (BCNN) for uncertainty estimation to improve the diagnostic performance. DeGrave, Janizek, and Lee^18^ demonstrated that recent Deep Learning systems for detecting COVID-19 from chest radiographs rely on confounding factors rather than medical pathology, creating an alarming situation in which the systems appear accurate but fail when tested in new hospitals. Pedrosa et al.^44^ found that deep learning systems for COVID-19 detection in chest radiography exhibited high dataset bias, leading to high performance in intradataset train-test scenarios but significantly lower performances in interdataset train-test scenarios. A systematic review by Roberts et al.^45^ found that machine learning models for COVID-19 detection using chest radiographs and CT scans were not of potential clinical use due to methodological flaws and biases.

### Multi-task learning

Multi-task learning (MTL) has emerged as a promising approach in Deep Learning to address the challenges of training neural networks for multiple tasks simultaneously. MTL aims to leverage shared knowledge across tasks to improve the generalization performance of the model. This approach has gained significant attention in various domains, including natural language processing, computer vision, and drug discovery^46,47^. By training a single neural network to perform multiple tasks, MTL offers several benefits, such as improved data efficiency, reduced overfitting through shared representations, and faster learning by leveraging auxiliary information^48^. Numerous studies have explored different methods and techniques for implementing MTL in deep neural networks^46,49,50^. These approaches have demonstrated the effectiveness of MTL in enhancing the model’s performance by exploiting task dependencies and learning shared representations^51,52^. However, designing an effective MTL framework requires careful consideration of task selection, model architecture, and optimization strategies^53^. In this paper, we build upon the existing literature and leverage MTL methods in Deep Learning, focusing on their introduction, theoretical foundations, and empirical effectiveness for COVID-19 detection from CXRs.

### Multi-task learning based COVID-19 detection on CXRs

There are several papers^54–56^ in the literature that employ the MTL paradigm for diagnosing COVID-19 from CXR images. COMiT-Net^54^ is an MTL framework that consists of tasks for lung segmentation and disease localization along with the primary task of healthy/unhealthy classification of CXR images . To exploit the rich amount of available CXR data for COVID-19 diagnosis, another MTL framework^55^ employs auxiliary task such as Pneumonia, Lung opacity and Pleural effusion detection from CXR images. However, MTL as a paradigm for addressing spurious correlations has not been explored in the literature. While previous papers have explored the use of MTL for COVID-19 detection, there is a lack of evidence demonstrating its effectiveness in developing robust models with reduced **PIP** and **OLS**. Additionally, the existing literature does not provide clarity on whether these Deep Learning architectures can be applied to implement an **AIDXA** system that supports radiologists and enhances the standard of care in rural areas. Therefore, in this paper, we conducted a comprehensive case study to evaluate the performance of MTL in the context of limited training data and investigate the stability of predictions as well as the location of activation maps associated with them when integrated with the **AIDXA** systems.

## Materials and methods

In this case study, the authors begin by addressing the challenges associated with popular architectures used for COVID-19 diagnosis when tested with CXR images transmitted through WhatsApp. To study the the impact of **PIP** and **OLS**, a new dataset called **WaCXR** dataset is created by passing the CXR images through WhatsApp messaging application. Subsequently, the paper discuss the architecture and the mathematical formulation of **COVIDMT**. **COVIDMT** is designed to learn from multiple datasets, which helps mitigate the limitations of existing architectures that are solely trained on COVID-19 datasets. By incorporating diverse data sources, **COVIDMT** aims to overcome the issues associated with single-dataset-based models.

### Discussion of existing CXR datasets

The COVID-Net^14^ dataset is a widely used publicly available collection of CXR images of COVID-19 infected patients. In addition to this dataset, the study also considers two other open source datasets: RSNA^57^, and NIH^58^. These datasets contain CXR images of patients infected with diseases other than COVID-19. The three datasets are extensively used in the existing literature for research purposes^14,32,59,60^. It is important to note that the collection of these datasets adhered to established protocols and guidelines, following the ethical regulations stipulated by the respective institutions. Informed consent was obtained from the participating subjects. Further information regarding these datasets can be found in^14,57,58^. For a comprehensive overview of the datasets, please refer to the summarized information presented in Table 1.

- *COVID-Net dataset*: The COVID-Net dataset^14^ contains CXR images of patients with COVID-19, pneumonia, and normal cases. The COVID-Net dataset is growing incrementally and has several versions released by the COVID-Net authors. The train and validation dataset used for this study is the train and validation set of the COVIDx3 CXR dataset. The test dataset used in this study consists of those CXR images in the COVIDx8 CXR dataset that are not present in the COVIDx3 CXR train and validation dataset. The training dataset consists of 5451 pneumonia, 7966 normal, and 110 COVID-19 CXR images. To increase the number of COVID-19 images in train dataset, upsampling is performed to match pneumonia images. The validation dataset includes 100 pneumonia, 100 normal, and 25 covid CXR images. The test dataset consists of 1750 normal and 4812 COVID-19 CXR images.
- *RSNA dataset*: The RSNA dataset^57^ consists of CXR images of patients infected with pneumonia and normal cases. Each CXR image contains bounding box information for patients with pneumonia, including the upper-left coordinates, width, and height of the bounding box. The RSNA dataset consists of 22638 CXR images in the training dataset and 7589 CXR images in the validation dataset, as provided by the authors.
- *NIH dataset*: The NIH CXR Dataset^58^ consists of 112,120 CXR images with disease labels from 30,805 unique patients. It is a multi-label dataset where patients can have normal CXR or be associated with one or more of the following 14 categories: Atelectasis, Cardiomegaly, Effusion, Infiltration, Mass, Nodule, Pneumonia, Pneumothorax, Consolidation, Edema, Emphysema, Fibrosis, Pleural-Thickening, and Hernia. For this study, only categories with more than 5.0% incidence in the dataset are considered. The number of CXR images in the train and validation sets for these selected categories is provided in Table 1.

### WaCXR: a dataset of WhatsApp compressed CXR images paired with original images

To understand the challenges faced by **AIDXA** systems due to compression caused by low-bandwidth messaging applications like WhatsApp, it is crucial to have a dataset that includes compressed images generated by these messaging applications. However, currently, there are no open-source datasets available with image pairs consisting of original CXR images and their corresponding compressed versions. To address this gap, we created a new in-house dataset using WhatsApp and the COVID-Net test dataset, called the WhatsApp CXR dataset (**WaCXR**  for short). To create the image pairs, all 6562 original COVID-Net test images in JPEG format were sequentially sent through WhatsApp to a fixed mobile number with a 60-s interval between each image. Once the WhatsApp compressed images were received on the mobile number, they were sorted based on their timestamps and paired with their respective original CXR images. In total, we generated 6562 image pairs, with 1750 pairs corresponding to normal scans and the remaining 4812 pairs corresponding to COVID-19 scans.

**Figure 3:**
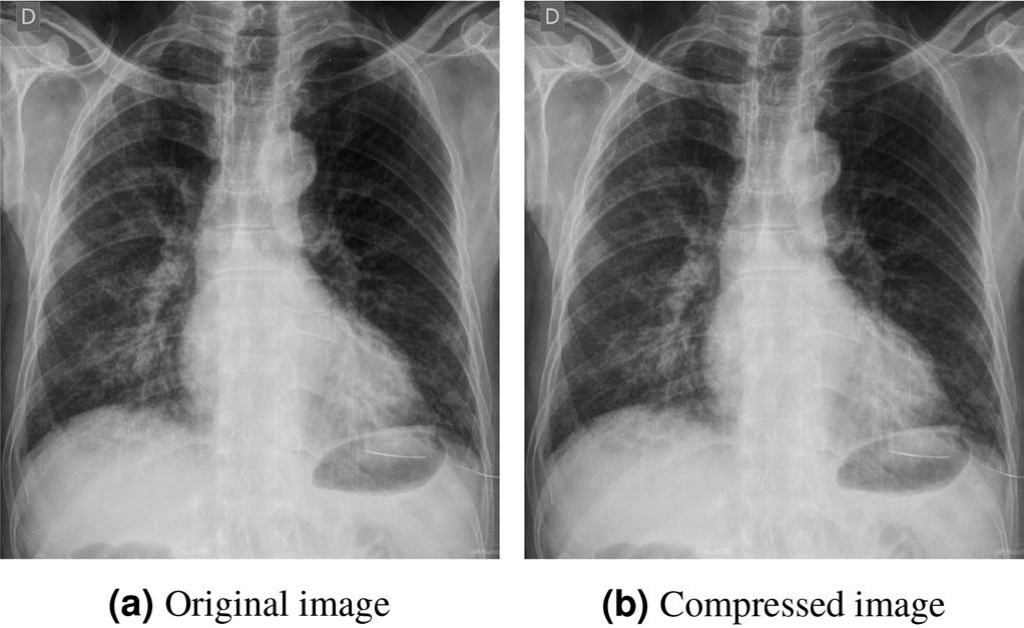
Illustration of a sample (**a**) original image and its corresponding (**b**) compressed image from **WaCXR** Dataset. Though they are visually similar, existing architectures such as ResNet-50 predicted (**a**) as +ve for COVID-19 and (**b**) as -ve for COVID-19.

At the conclusion of the experimentation, it was observed that the WhatsApp compressed CXR images only occupied 351 MB of memory, whereas the original COVID-Net test data occupied 6.7 GB. This indicates a compression factor of 95% achieved by WhatsApp. However, this compression can alter the intricate intensity values of the images and potentially lead to inaccurate diagnoses if the models are trained solely on high-resolution images. Figure 3 presents a sample image from the COVID-Net test dataset alongside its corresponding compressed counterpart. The existing state-of-the-art (SOTA) models predicted different diagnoses for the high-resolution (original) image compared to the low-resolution (compressed) images. Additionally, the activation maps used for localization showed significant differences, often highlighting non-lung regions. Clearly, this calls for more research and thus, this dataset is open-sourced along with the paper to encourage research in this area.

**Table 1:** Distribution of different abnormalities of CXR images in COVID-Net, RSNA and NIH datasets.

Dataset	Categories	Train-set size	Validation-set size	Test-set size
COVID-Net	Normal	7966	100	1750
	Pneumonia	5451	100	–
	COVID-19	110	25	4812
RSNA	Pneumonia	7134	2421	–
	Non-Pneumonia	15,504	5168	–
NIH	Infiltration	13,914	2018	–
	Effusion	9261	1292	–
	Atelectasis	7996	1119	–
	Nodule	4375	625	–
	Normal	5000	800	–

### Challenges in diagnosing Xray images received through smart phones

As part of the case study aimed at understanding the challenges associated with **AIDXA** in diagnosing CXR images, a paired dataset called **WaCXR** Dataset was created. This dataset was used to experiment with five state-of-the-art Deep Neural Networks for the diagnosis of COVID-19 from CXR images. The case study highlights two problems introduced in this section: *prediction instability* (**PIP**) and *out-of-lung saliency* (**OLS**) that arise in state-of-the-art Deep Neural Networks when diagnosing CXR images received through smartphone applications like WhatsApp.

#### Deep learning architectures for COVID-19 diagnosis

This study investigates several popular state-of-the-art Deep Neural Networks (ResNeXt-50^11^, ResNet-50^10^, XceptionNet^12^, VGG-19^13^, and COVID-Net^14^) for COVID-19 prediction in order to understand the challenges of deploying mobile phone-driven diagnosis. Each architectures was trained using the COVID-Net dataset following standard practices. The study highlights the issues of **PIP** and **OLS** when these architectures are used to predict labels for images in the **WaCXR** dataset. For detailed information on data preprocessing, please refer to the Experiments section.

### The prediction instability problem

**PIP** of a predictive model is defined as the probability of disagreement between the predictions on a randomly perturbed instance and the true instance. Let us consider a deep learning model M that classifies a CXR image into COVID-19 positive or COVID-19 negative. One would prefer that M gives the same prediction both on the original CXR image and the image transmitted through a **AIDXA**. Since the images are different, one can view the transmitted image as a random transformation R of the original CXR. Formally for a model $\texttt{M}: {{\mathscr {X}}} \rightarrow {{\mathscr {Y}}}$

1 $$\begin{aligned} PIP(\texttt{M}) = P{\textrm{rob}}\left( y(x; \texttt{M}) \ne y(\texttt{R}(x);\texttt{M})\right) \end{aligned}$$

where $x \in {{\mathscr {X}}}$  and $\texttt{R}$ is a random perturbation of the instance.

Consider a dataset $D =\{X_i, \texttt{R}(X_i) \}_{i=1}^N$ consisting of *N* pairs of original CXR image ($X_i$), and $\texttt{R}(X_i)$ denotes **AIDXA** compressed image of $X_i$. The estimate of *PI Score* due to image perturbation by **AIDXA** can be now defined as the fraction of pairs where the predictions differed expressed as percentage.

2 $$\begin{aligned} PI\,Score(\texttt{M}; D) = \frac{1}{N}\left( \sum _{\{X_i, \texttt{R}(X_i)\} \in D } {{\textbf{I}}}\left( y(X_i; \texttt{M})\right) \ne {{\textbf{I}}}\left( y(\textbf{R}(X_i);\texttt{M}) \right) \right) \times 100 \end{aligned}$$

#### PI score results on SOTA

The *PI Score*, as shown in Table 2 is calculated for the state-of-the-art Deep Learning Networks^10–13^ using the **WaCXR** dataset. The results clearly indicate that all models exhibit *prediction instability*. Considering the significance of accurate prediction outcomes, it is quite alarming that in certain models, the level of prediction instability can reach upto $10\%$.

**Table 2:** PI score for different techniques on **WaCXR** dataset. From the table, it is evident that the current SOTA face PIP.

Model	PI score
XceptionNet^12^	7.12
VGG-19^13^	4.82
ResNet-50^10^	7.5
ResNeXt-50^11^	6.28
COVIDNet-CXR-Small^14^	4.36
COVIDNet-CXR3-B^14^	4.49
COVIDNet-CXR4-B^14^	11.71
COVIDNet-CXR4-C^14^	4.60

### Problem of spurious features:out-of-lung saliency

It is now widely recognized that Deep Networks’ predictions can be heavily influenced by spurious features, especially in the case of CXR images^18,37^. Such predictions are undesirable, particularly in the context of medical diagnosis, as they result in unexplainable outcomes. Central to this paper is the intuition that a model designed to identify lung ailments from CXR images suffers from the issue of spurious features if it predominantly relies on pixels located outside the lung region for its predictions. To address this, saliency map-based techniques, such as GradCAM^61^, are employed to attribute features to predictions. In this study, these saliency maps are utilized to establish a quantitative measure for assessing the impact of spurious features.

Given a Deep Learning Model for diagnosing COVID-19 from CXR images, *out-of-lung saliency* (**OLS**) is defined as the correlation between attributed pixels outside the lung region and model prediction. Existing literature^18^ examines the effect of **OLS** on the model’s decision through examples of saliency maps on individual images, emphasizing the need to quantify the effect of **OLS** at a population level. To address this, the paper proposes a metric called the *Out of Lung Saliency Score (OLS Score)* to aggregate **OLS** across a population.

#### Out of lung saliency score (OLS score)

This paper introduces the *OLS Score* as a means to quantify the impact of **OLS** on the diagnosis made by state-of-the-art models. The *OLS Score* is derived using *Intersection Over Lung-Region (IOL)*, which measures the extent of **OLS** on each CXR image in the population. *IOL* provides a quantification of the effect of **OLS** on the CXR image level, while the *OLS Score* quantifies the overall effect of **OLS** on a population level.

Let $heatmap\text {-}region(y(x;\texttt{M}))$ indicate the set of pixels generated by GradCAM^61^ as a saliency map of model M when acting on a CXR image *x* and producing a prediction *y*. To identify spurious features, lung segmentation techniques are utilized to determine pixels that are both within the lung region and heatmap region. Let $lung\text {-}region(x)$ denote the pixels indicating lung region in *x*. In this work, lung-region is identified using a segmentation model^62^.

*IOL* is defined as the ratio of the number of pixels in the intersection of heatmap region and the lung region of a CXR image to the total number of pixels in the heatmap region. For a given a CXR image *x*, which is represented by its pixels and intensity values, define

3 $$\begin{aligned} IOL(x;\texttt{M}) = \frac{{\texttt{Pix}}\left( heatmap\text {-}region(y(x; \texttt{M})) \cap lung\text {-}region(x)\right) }{\texttt{Pix}\left( heatmap\text {-}region(y(x; \texttt{M}))\right) } \end{aligned}$$

where Pix(*R*(*x*)) counts the number of Pixels in region *R*(*x*), a subset of the pixels in the original image *x*. The IOL value ranges from 0 to 1, where an IOL of 0 indicates that the prediction relies entirely on pixels outside the lung region (indicating spurious correlations), while an IOL of 1 indicates that the prediction is based solely on pixels within the lung region. The OLS Score of a model M on a dataset *D* consisting of *N* images is then defined as the percentage of images in *D* with an *IOL* value less than $\eta$.

4 $$\begin{aligned} OLS \, Score_{\eta }(D; \texttt{M}) = \frac{1}{N}\left( \sum _{x \in D} IOL(x; \texttt{M}) < \eta \right) \times 100 \end{aligned}$$

The value of $\eta$ in this paper is set to 0.4. For brevity, we will exclude $\eta$ from the subscript. The OLS Score measures the number of images that have a low *IOL*, indicating an *IOL* value less than $\eta$. This quantifies the extent to which spurious effects, such as pixels outside the lung region, contribute significantly to the outcome *y*. A high OLS Score indicates that a majority of the data is affected by out-of-lung saliency, while a low OLS Score suggests a lesser impact of such spurious features.

**Figure 4:**
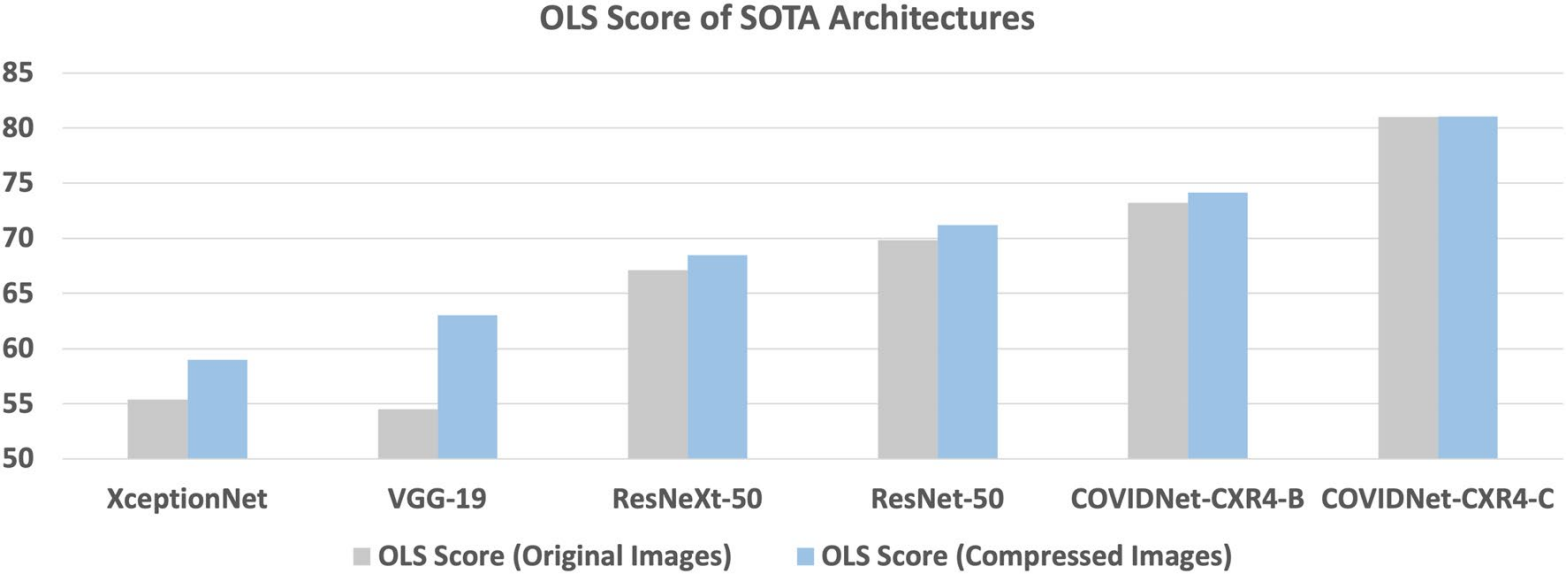
OLS Score results of SOTA models^10–14^ on **WaCXR** Dataset. From the figure, it is evident that the current SOTA face significant **OLS** when the images are perturbed with WhatsApp compression.

#### OLS score results on SOTA

 The *OLS Score* of the dataset, as defined in the previous paragraph, is analyzed on several state-of-the-art Deep Learning Networks using the **WaCXR** dataset. In this study, heatmaps of CXR images were generated using GradCAM^61^ for each state-of-the-art models. For each CXR image, the lung region was identified using a U-Net^62^. This was used to compute the *OLS Score*. The results (Fig. 4), clearly show that state-of-the-art models rely on features outside the lungs as crucial factors in determining whether a CXR image belongs to a patient infected with COVID-19. Moreover, the problem of **OLS** is further exacerbated in CXR images compressed by WhatsApp. The state-of-the-art models experimented with have an average *OLS Score* of $66\%$ for original images, while it increases to 70% for the WhatsApp images. Notably, the COVID-Net models used in this study exhibit an *OLS Score* as high as 70%. These findings indicate that CXR images diagnosed using state-of-the-art models are prone to the issue of **OLS**, as the localization map primarily highlights pixels located outside the lung region. This could be attributed to factors such as corner labels, soft tissue, and scan tool configurations present in the CXR image.

### MTL for smartphone-based diagnosis of COVID-19 from CXR images

In this case study, we have examined the challenges faced by Smartphone-based **AIDXA** applications that were launched for rapid triaging of infected patients during the COVID-19 pandemic. The case study utilizes CXR images transmitted via low-bandwidth messaging applications such as WhatsApp for AI-based diagnosis of COVID-19. The experimental evidence presented in the case study provides compelling support for the assertion that state-of-the-art models are plagued by the issues of **PIP** and **OLS**. Consequently, these models are not suitable for seamless integration with messaging applications like WhatsApp for diagnosing using CXR images. Among the state-of-the-art models investigated, ResNet, ResNeXt and COVIDNet-CXR4-B are particularly affected by **PIP** and **OLS**. It remains an open question as to what mitigation strategies could effectively address these challenges. In light of these findings, the case study proceeds to explore multi-task learning (MTL) as a potential strategy to address these issues.

### **COVIDMT**: MTL network for COVID-19 diagnosis

**Figure 5:**
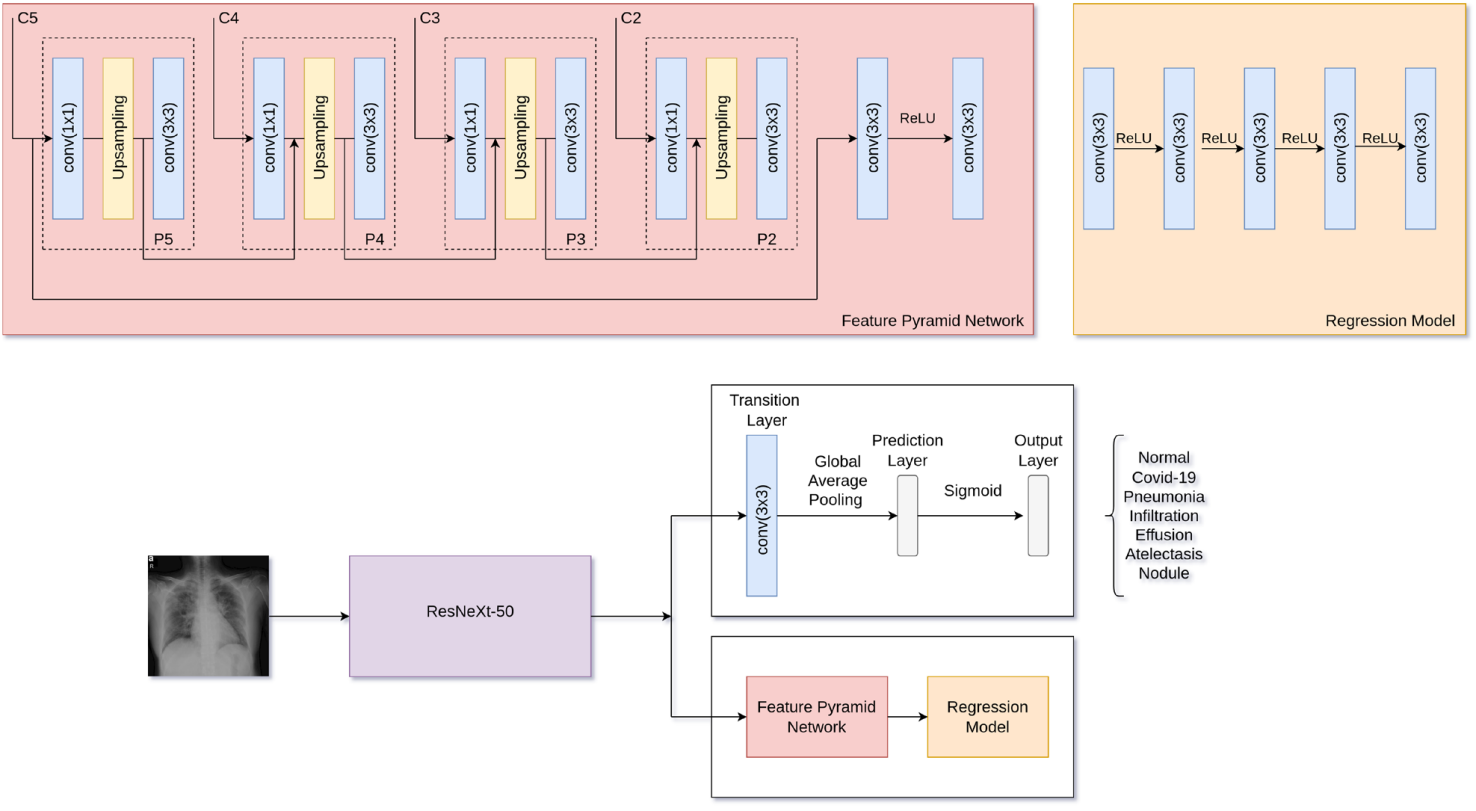
**COVIDMT** model architecture with ResNeXt-50 as base network.

MTL or models that learn multiple tasks simultaneously, have been shown to outperform single-task models. This section introduce the architecture of **COVIDMT**, a multi-task Deep Learning Network for COVID-19 diagnosis. **COVIDMT** is built on top of a state-of-the-art Deep Learning Network known as a *base network*. The *base network* is initialized with Imagenet weights to enable *transfer learning*, thereby maximizing the performance of **COVIDMT** model on the target domain. In **COVIDMT**, the base-network is shared by all tasks. The feature vector from base-network is utilized by task-specific networks optimized for CXR classification and bounding-box regression tasks. The architecture of **COVIDMT** is depicted in Fig. 5, and comprises the following main components,

- *Base network*: The base network is a state-of-the-art neural network with the last layer removed. In **COVIDMT**, all tasks share the same *base networks*. The study experiments **COVIDMT** with different *base networks*, including ResNeXt^11^ and ResNet^10^. Pre-training the *base networks* with ImageNet dataset facilitates *transfer learning* within the multi-task learning architecture.
- *Classification head*: The classification head performs multi-label classification, assigning a CXR image to one or multiple classes simultaneously. It consists of a transition layer of convolutions (CNN), followed by Global average pooling (GAP), and a Prediction layer of a fully connected neural network.
- *Bounding box detection head*: The bounding box detection head identifies bounding boxes in an image, if present. The bounding box regression sub-network incorporates the Feature Pyramid Network (FPN)^63^, followed by the Box Regression subnet for bounding box localization^64^. The FPN consists of a sequence of convolution, upsample and convolution operations repeated three times, followed by four convolution layers. The Box Regression Subnet comprises four sequential layers of convolution followed by ReLU, with a final convolution layer. The bounding box head is designed to detect visual signal of pneumonia in the RSNA dataset, and the use of FPN for bounding box detection yields excellent performance on the RSNA dataset^65^.

#### Mathematical formulation of **COVIDMT**

Consider COVID-19 detection problem from CXR images using **COVIDMT** with *T* tasks over an input space *X* and a collection of tasks spaces $\{Y\}_{t=1}^{T}$. The hypothesis class of the problem, denoted as *H*, can be defined as $H = \{F \}_{t=1}^{T} \circ G$. $G = \{g: {{\textbf{x}}}\rightarrow R^k \}$, is a set of *base network* and *k* is the dimension of representation space of the feature vector. $F_{t=1}^{T}$ $=\{ f^{t}: {\mathbb {R}}^k \rightarrow y\}_{t=1}^T$ is a set of task-specific networks. *g* is the representation shared across different tasks while $f^t$ is task specific. $H = \{ h= \{f^t(g(\cdot ) \}_{t=1}^{T} : {{\textbf{x}}}\rightarrow \{ y\}_{t=1}^T\}$. Assume that each task *t* has $n_t$ samples. $\{{{\textbf{x}}}_i^t, y_i^t \}_{i=1}^{n_t} = D_t$, where $D_{t}$ is the training dataset for task $t=\{1,..., T\}$. The loss function for task *t* is defined as $l^t: y \times y \rightarrow [0,1]$.

**COVIDMT** for COVID-19 detection is a six-task MTL network. Table 3 provides information about the tasks, datasets used for each task, the type of task, and the corresponding loss function. For the multi-label classification task, the sigmoid cross-entropy loss function is used as shown in Table 3. For bounding box regression task, loss is given by regularized least squares objective^66^ as shown in Table 3, where $y_{i}$ represents the pixel coordinates of the bounding box in image ${{\textbf{x}}}_i$ and $R(f^{bbox})$ is the L2 regularization of weights in the Box regression subnet.

**Table 3:** Summary of different tasks along with datasets, type of task and loss functions.

Task	Dataset	Type of task	Loss function ($L_{D_t}^{t}({{\textbf{x}}},y)$)
COVID-19	COVID-Net	Classification	$- \left[ y \log (f^t(g({{\textbf{x}}}))) + (1 - y) \log (1- f^t(g({{\textbf{x}}}))) \right]$
Infilteration	NIH		
Effusion	NIH		
Atelectasis	NIH		
Nodule	NIH		
Bounding box detection	RSNA	Regression	$\left[ (f^{bbox}(g({{\textbf{x}}})) - y)^2 + R(f^{bbox}) \right]$

The empirical loss for task *t* is defined as $L^t_{D_t} = \frac{1}{n_t} \sum _{i=1}^{n_t} l^t (f^t(g({{\textbf{x}}}_i^t)), y_i^t)$. Let $T_{CLS}$ be the number of classification tasks and $T_{REG}$ be the number of regression tasks such as $T = T_{CLS} + T_{REG}$ (see Table  3). The empirical task-averaged error is defined as follows,

5 $$\begin{aligned} L_D = \frac{1}{T} \sum _{t=1}^{T} L_{D_t}^{t} \end{aligned}$$

## Evaluation of **COVIDMT** model

As previously observed, the case study reveals the challenges of **PIP** and **OLS** associated with state-of-the-art models integrated with low-bandwidth messaging applications for COVID-19 diagnosis. Furthermore, this case study demonstrates that MTL serves as an effective defense against the problems of **PIP** and **OLS**. This section examines the effectiveness of MTL model **COVIDMT** when compared with state-of-the-art models to mitigate the issue of **PIP** and **OLS**. The analysis is conducted using the **WaCXR** dataset, which consists of paired original and WhatsApp compressed CXR images. This section commences with a discussion of the data preprocessing and implementation details of **COVIDMT**. As stated previously, *PI Score* and *OLS Score* are employed to analyze the effect of **PIP** and **OLS** in Deep Learning Models. In this section, the *PI Score* and *OLS Score* of **COVIDMT** are evaluated to analyze their impact on COVID-19 diagnosis.

### Data preprocessing

The input image dimension is 512 * 512, and pixel values are normalized. Since there are fewer COVID-19 CXR images, this study up-sample COVID-19 images and apply the following data augmentation techniques: translation ($\pm 10\%$ in x and y directions), rotation ($\pm 10$°), horizontal flip, zoom ($\pm 15\%$), and intensity shift ($\pm 10\%$).

### Implementation details

**COVIDMT** model is implemented using Pytorch. The model is trained using the Adam optimizer with an initial learning rate of 2e−4. A multistepLR scheduler with milestones at epochs 8, 16, and 20 is employed. In each iteration, the batch size for each learning task, corresponding to COVID-Net, RSNA^57^, and NIH dataset^58^ is 4 and the model is trained for 25 epochs. The values of these parameters were determined through hyperparameter tuning to maximize the model’s performance on the validation dataset.

**Figure 6:**
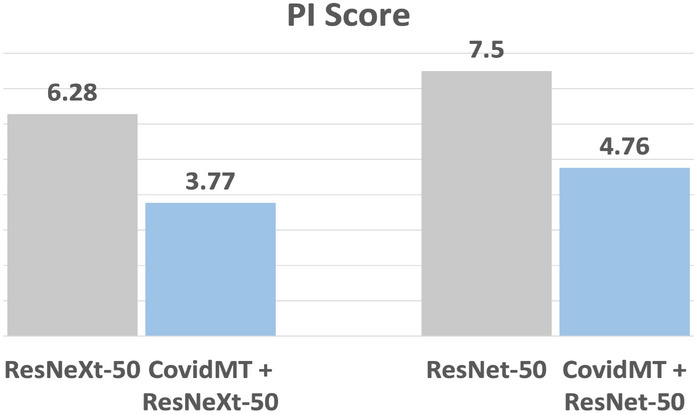
Results showing a reduction of 40% in prediction instability problem with **COVIDMT** on **WaCXR** dataset.

### COVIDMT for reducing prediction instability and out-of-lung saliency

As shown in Table 2 and Fig. 4, this study evaluates the performance of five state-of-the-art models such as ResNet, ResNeXt, VGG, Xception and COVIDNet, in terms of **PIP** and **OLS**. Among these state-of-the-art models, ResNeXt, ResNet and COVIDNet-CXR4-B resulted in the least performance in terms of **PIP** and **OLS** as observed from Table 2 and Fig. 4. However, the implementation of COVIDNet-CXR4-B architecture is not openly available. Hence, the remaining two underperforming architectures ResNet and ResNeXt are selected for the case study to demonstrate the benefits of **COVIDMT**.

**Figure 7:**
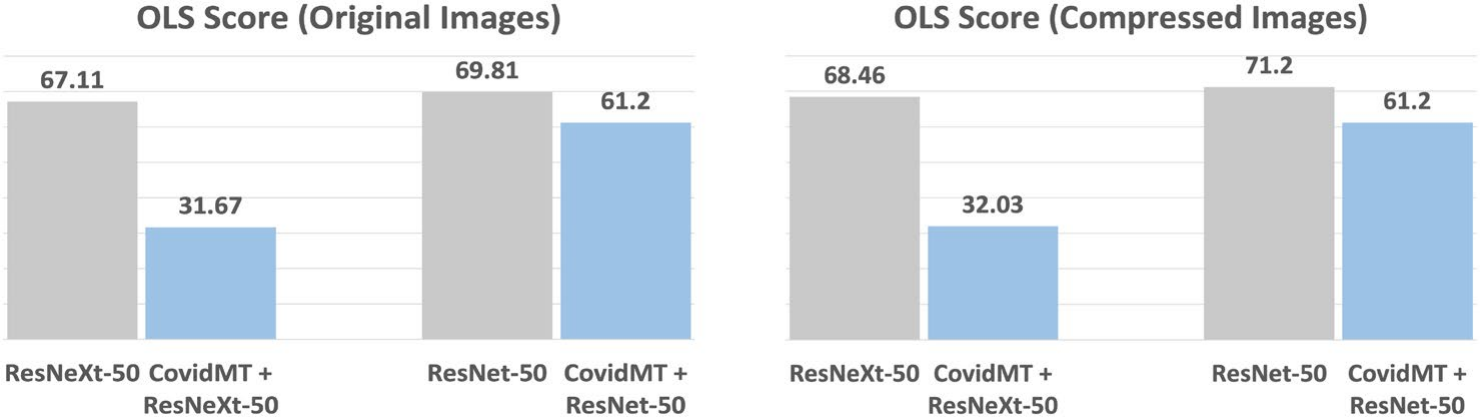
Results showing a reduction of 35% in out-of-lung saliency with **COVIDMT** on **WaCXR** Dataset.

**Figure 8:**
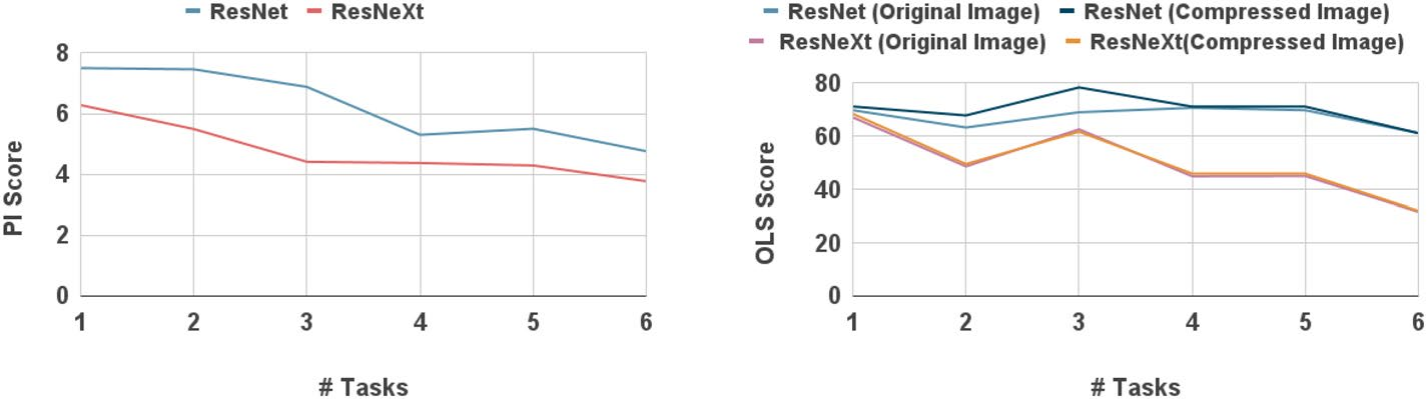
Results showing that MTL with six tasks has significantly less **PIP** and **OLS** when compared to MTL with fewer tasks on **WaCXR** Dataset.

The *PI Score* and *OLS Score* of base-models and **COVIDMT** applied to the base-models are presented in Figs. 6, 7 and 8, respectively. The results (Fig. 6), clearly indicate that **COVIDMT** reduces the *PI Score* of the base models by 40%. The average *PI Score* of ResNet and ResNeXt is 7%, whereas the average *PI Score* of **COVIDMT** models is 4.2%.

To compute the *OLS Score*, heatmaps of CXR images are generated using GradCAM^61^ and the lung region of each CXR image is identified using a U-Net^62^. U-Net^62^ is a convolutional neural network developed for biomedical image segmentation tasks. In this case study, U-Net^62^ is utilized to segment the lung region in each CXR image, which serves as the region of interest (ROI). The lung segmentation using U-Net^62^ is crucial as it allows to evaluate whether the model’s activation map falls within the lung regions or extends into non-lung regions. The lung region extracted by U-Net from the CXR image along with the heatmap region is utilized for the calculation of the IOL Score, which is then used to evaluate OLS Score in **WaCXR** Dataset.

**Figure 9:**
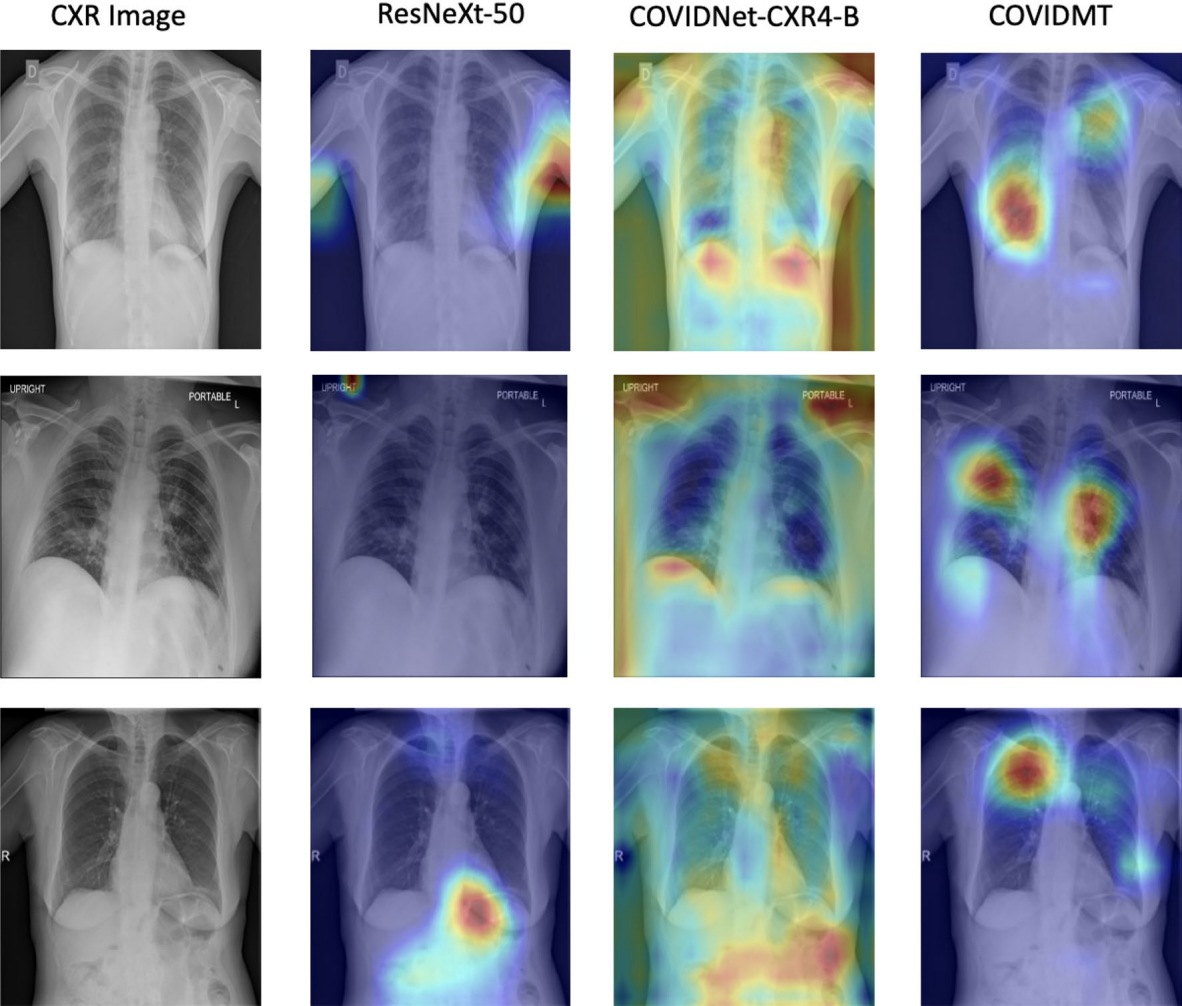
Exemplified COVID-19 localization with Vanilla ResNeXt-50^11^, SOTA COVID-Net^14^ Model and ResNeXt-50 with **COVIDMT**. The visualization of **COVIDMT** shows better localization with heat maps distributed majorly inside the lung region when compared to SOTA COVID-Net^14^ Model and Vanilla ResNeXt-50^11^, where heat maps are majorly distributed outside the lung region.

The results in Fig. 7 demonstrate that **COVIDMT** reduces the OLS Score of the base models by 35% for both original and compressed images, showcasing the effectiveness of multi-task learning. Specifically, the *OLS Score* of ResNeXt decreases from 67.11 to 31.67% for original images and 68% to 32% for compressed images when **COVIDMT** is applied. Example cases of from the **WaCXR** Dataset, along with saliency maps generated by the baselines and **COVIDMT** is presented in Fig. 9. It is evident that state-of-the-art Deep Networks heavily rely on **OLS** for COVID-19 diagnosis, while **COVIDMT** mitigates the impact of **OLS**.

To further understand the effect of the number of tasks on **PIP** and **OLS**, an ablation study is conducted. In this study, an MTL model is trained using the COVID-Net dataset as the primary task, and other tasks (as listed in Table 3) are incrementally added to train MTL models with upto six tasks. The primary task is the classification of CXR images in the COVID-Net dataset, and the subsequent tasks include bounding box detection using the RSNA dataset and classification tasks using the NIH dataset for infiltration, effusion, atelectasis, and nodule categories respectively. Each MTL model is evaluated using the **WaCXR** dataset. The result of the ablation study for **COVIDMT** with ResNet and ResNeXt as base models are presented in Fig. 8. It is evident from the results that as the number of tasks increases, the **PIP** of the MTL model decreases. This reduction can be attributed to the enhanced generalizability achieved through multi-task learning. Moreover, the **OLS** of the six-task MTL model is significantly lower compared to MTL models with fewer tasks. Overall, the ablation study demonstrates the positive impact of incorporating multiple tasks in the MTL framework. It contributes to reducing **PIP** and **OLS**, thereby improving the robustness and performance of the **COVIDMT** model.

## Discussion

Adopting AI-based Medical Diagnosis systems integrated with WhatsApp for COVID-19 poses certain challenges. One of the major challenges is accessibility, particularly in LMICs, where poor network connections hinder medical practitioners’ access to these automated services. To address this accessibility gap, smartphones and low-bandwidth messaging services like WhatsApp have been considered as viable solutions. However, it is crucial for these AI services to maintain high classification performance. This study reveals that AI-based medical diagnosis systems integrated with WhatsApp for image transfer face issues related to **PIP** and **OLS**, which are crucial requirements for any Medical Diagnosis system. While recent research works have highlighted the impact of spurious correlations in model predictions using CXR images, they have not adequately quantified and addressed the confounding effect between model prediction and pathology.

This study proposes metrics to quantify the effect of **PIP** and **OLS** resulting from the spurious confounding between model prediction and pathology. Our investigations demonstrate that the proposed MTL framework helps build robust Deep Learning Models by mitigating **PIP** and **OLS**. While recent works^67,68^ have shown that MTL is robust aganist adversarial perturbations of input image, the problem of **PIP** and **OLS** in the context of image perturbation has yet to be studied in the literature.

As discussed in the paper, the image compression used by WhatsApp for sharing images can lead to the misclassification of positive (+ve) and negative (−ve) classes, resulting in **PIP**. The proposed *PI Score* quantifies the effect of **PIP** in the models, and the results indicate that state-of-the-art models can have a high *PI Score* of upto 10%. The MTL strategy proposed in this study, with access only to high-resolution CXR images, is robust to **PIP**, leading to a 40% reduction in the *PI Score*.

Another factor that hinders the adoption of computer-aided platforms is the lack of explainability in AI model predictions^69^. While existing literature has addressed the issue of explainability for COVID-19 detection by providing saliency maps of a few CXR images, it has been observed that most saliency maps generated by existing models focus outside the lung region^45^, resulting in spurious correlations. To address this, the study introduces the *OLS Score* (Eq. 4), which quantifies the confounding effect. The MTL framework significantly reduces the *OLS Score* by 35% and improves the classification performance of vanilla networks such as ResNet and ResNeXt, achieving state-of-the-art results. The MTL framework learned the contextual information of lung regions from the wide range of non-COVID-19 CXR images during the training. This is in contrast with learning solely from the limited available COVID-19 data, which may lead to spurious correlations.

## Conclusion

The COVID-19 pandemic has brought to light the vulnerabilities within our current healthcare ecosystem. Smartphone-based AI-aided Diagnosis of X-ray images through Apps (**AIDXA**) has emerged as an effective solution to bridge the accessibility and expertise gaps in rural areas of LMICs. However, it is crucial to assess the technical challenges of these **AIDXA** systems in terms of their predictions. As there are no existing quantitative descriptors, new metrics such as **PIP** and **OLS** are proposed in this paper. The experiments presented in this paper demonstrate the presence of **PIP** and **OLS** issues in the existing state-of-the-art deep learning architectures when integrated with **AIDXA** systems using WhatsApp as a communication medium. To address these issues, this paper proposes the use of a multi-task learning framework for COVID-19 detection, known as **COVIDMT**. In future research, it would be interesting to explore the challenges of **PIP** and **OLS** in relation to different abnormalities and imaging modalities. Additionally, investigating the potential of a multi-task learning framework to address these issues could be a promising direction for further exploration.

## Data Availability

The datasets generated and/or analysed during the current study are available in the **WaCXR** Dataset repository, https://github.com/mariamma/WACXRDataset.
